# The limitations to our understanding of peer review

**DOI:** 10.1186/s41073-020-00092-1

**Published:** 2020-04-30

**Authors:** Jonathan P. Tennant, Tony Ross-Hellauer

**Affiliations:** 1 Institute for Globally Distributed Open Research and Education, Gianyar, Bali Indonesia; 2 Graz University of Technology & Know Center GmbH, Graz, Austria

**Keywords:** Peer review studies, Quality control, Quality assurance, Scholarly communication, Open peer review, Scholarly publishing, Reproducibility, Research impact

## Abstract

Peer review is embedded in the core of our knowledge generation systems, perceived as a method for establishing quality or scholarly legitimacy for research, while also often distributing academic prestige and standing on individuals. Despite its critical importance, it curiously remains poorly understood in a number of dimensions. In order to address this, we have analysed peer review to assess where the major gaps in our theoretical and empirical understanding of it lie. We identify core themes including editorial responsibility, the subjectivity and bias of reviewers, the function and quality of peer review, and the social and epistemic implications of peer review. The high-priority gaps are focused around increased accountability and justification in decision-making processes for editors and developing a deeper, empirical understanding of the social impact of peer review. Addressing this at the bare minimum will require the design of a consensus for a minimal set of standards for what constitutes peer review, and the development of a shared data infrastructure to support this. Such a field requires sustained funding and commitment from publishers and research funders, who both have a commitment to uphold the integrity of the published scholarly record. We use this to present a guide for the future of peer review, and the development of a new research discipline based on the study of peer review.

## Introduction

Peer review is a ubiquitous element of scholarly research quality assurance and assessment. It forms a critical part of a research and development enterprise that annually invests $2 trillion US dollars (USD) globally [1] and produces more than 3 million peer-reviewed research articles [2]. As an institutional norm governing scientific legitimacy, it plays a central role in defining the hierarchical structure of higher education and academia [3]. Now, publication of peer-reviewed journal articles plays a pivotal role in research careers, conferring academic prestige and scholarly legitimacy upon research and individuals [4]. In spite of this crucial role it plays, peer review remains critically poorly understood in its function and efficacy, yet almost universally highly regarded [5–11].

As a core component of our immense scholarship system, peer review is routinely and widely criticised [12–14]. Much ink has been spilled on highly cited and widely circulated editorials either criticising or championing peer review [15–21]. A number of small- to medium-scale population-level studies have investigated various aspects of peer review’s functionality (see [12, 22, 23] for summaries); yet the reality is that there remain major gaps in our theoretical and empirical understanding of it. Research on peer review is not particularly well-developed, especially as part of the broader issue of research integrity; often produces conflicting, overlapping or inconclusive results depending on scale and scope; and seems to suffer from similar biases to much of the rest of the scholarly literature [8].

As such, there is a real danger that advocates of reform in peer review do not always appreciate the often-limited scope in our general understanding of the ideology and practice of peer review. Ill-informed generalisations are abound, for example, the oft-heard ‘peer review is broken’ rhetoric [24, 25], compared with those who herald it as a ‘golden standard’. Peer review is also often taken as a hallmark of ‘quality’, however, despite the acknowledgement that it is also an incredibly diverse and multi-modal process. The tensions between these viewpoints create a strange dissonant rationale, that peer review is uniform and ‘the best that we have’, yet also flawed, often without fully appreciating the complexity and history of the process [26–29]. Consequently, debates around peer review seem to have become quite polarised; it either remains virtually untouchable, and often dogmatically so, as a deeply embedded structure within scholarly communication, or is something fatally corrupted and to be abandoned in toto. On the one hand, criticisms levied towards peer review can be seen as challenging scientific legitimacy and authority and therefore creates resistance towards developing a more nuanced and detailed understanding of it, both in terms of practice and theory. On the other hand, calls for radical reforms risk throwing out the baby with the water so imply systematic understanding of peer review as irrelevant.

This makes inter- and intra-discipline and systematic comparisons about peer review particularly problematic, especially at a time when substantial reform is happening across the wider scholarly communication landscape. The diversity of stakeholders engaging with peer review is now increasing with the ongoing changes around ‘Open Scholarship’; for example, policymakers, think-tanks, research funders and technologists are increasingly concerned about the state of the art in research and its communication and role in wider society, for example, regarding the United Nations Sustainable Development Goals. In this context, developing a collective empirical and theoretical understanding of the function and limitations of peer review is of paramount importance. Specifically available funding for such research is also almost entirely absent, with exceptions such as the European Commission-funded PEERE initiative [30, 31]. This is especially so when compared to relatively rapidly accumulating attention for research reproducibility [32–36], now with calls specifically for research on reproducibility (e.g. via the Association for Psychological Science or the Dutch Research Council). There is now an imperative for the quantitative analysis of peer review as a critical and interdisciplinary field of study [9, 31, 37–39].

This article aims to better explore and demarcate the gaps in our understanding of peer review to help guide future exploration of this critical part of our knowledge infrastructure. Our primary emphasis is to provide recommendations for future research based around the need for a rigorous and coordinated programme focused on a new multi-disciplinary field of Peer Review Studies. We provide a roadmap that highlights the difficulty and priority levels for each of these recommendations. This study complements ongoing and recent work in this area around strengthening the principles and practices for peer review across stakeholders [40].

## Methods

To identify gaps in our knowledge, we identified a number of core themes around peer review and peer review research. We then identified relevant literature, primarily based around recent meta-reviews and syntheses to identify the things that we do know about peer review. We then ‘inverted’ this knowledge and iteratively worked through each core theme to identify what we do not know at varying levels in a semi-systematic way. Part of this involved discussions with many colleagues, both in formal and informal settings, which greatly helped to shape our understanding of this project, highlight relevant literature, as well as identify the many gaps we had personally overlooked. We acknowledge that this might not have been sufficient to identify all potential gaps, which are potentially vast, but it should provide a suitable method for identifying major themes of interest for the main stakeholder groups.

Within these themes, we have attempted to make clear those things about peer review which are in principle (and may likely remain) obscure, as well as those things which are in principle knowable but currently obscure practically due to a lack of data or prior attention. The consequence of this structural interrogation is that we can begin to identify strategic research priorities and recommendations for the future of peer review research at a meta-level [40]. The assessments of priority and difficulty level are largely subjective and based on our understanding of issues surrounding data availability and their potential influence on the field of peer review. These research topics can also be used to determine what the optimal models of peer review might be between different journals, demographics and disciplines and interrogate what ‘quality’ means under different circumstances. Data sources here can include those obtained through journals/publishers sharing their data, empirical field studies, studying historical archives, interviews or surveys with authors, editors and reviewers or randomised controlled trials [22, 38, 41–44].

## Results and discussion

In this section, we will discuss the limits to our knowledge of peer review in a general, interdisciplinary fashion. We focus on a number of core themes. First, we discuss the role of editors; issues surrounding their accountability, biases and conflicts of interest; and the impact this can have on their decision-making processes. Second, we discuss the roles of peer reviewers themselves, including the impacts of blinding, as well as notions of expertise in what constitutes a ‘peer’. Third, we discuss the intended purpose and function of peer review and whether it actually upholds these things as a quality control mechanism. Fourth, we consider the social and epistemic consequences of peer review. Finally, we discuss some of the ongoing innovations around [open] peer review tools and services and the impacts that these might have.

### Roles of editors in peer review

Editors have a non-uniform and heterogeneous set of roles across journals, typically focused in some way around decision-making processes. Here, when we refer to ‘an editor’, we mean someone in such a position of authority at a journal, including editors-in-chief, managing editors, associate editors and all similar roles. Typically, by focusing on a binary outcome for articles (i.e. reject or accept), editorial peer review has become more of a judicial role than a critical examination [45], as the focus becomes more about the decision rather than the process leading to that decision. Justifications or criteria for editorial rejections (either ‘desk’ rejections or following peer review), and decisions, overall, are rarely given or automated, and poorly known despite perhaps being one of the most frustrating elements of the scholarly publishing process. It is rarely explicitly known whether journals send all submissions out for peer review or are selective in some way, for example, based on the scope of the journal and the perceived fit of articles. There are almost no studies regarding the nature of editorial comments and how these might differ from, or complement, respective reviewer comments. An analysis of these issues across a wide range of journals and disciplines would provide insight into one of the most important components of scholarly research.

We currently only have patchy insight into factors such as the number of times a paper might have been rejected before final acceptance, and further critical insight is needed into the general study of acceptance rates [46–48]. This is especially so as authors will very often search for another journal or venue to have their paper published when rejected by a single journal, which has important implications for journal-based evaluation systems. Limited available evidence suggests that a relatively small pool of researchers does the majority of the reviewing work [49–51]. This raises questions about how often editors elect to use ‘good’ or critical reviewers without exhausting or overworking them, and the potential consequences this might have on professional or personal relationships between the different parties and their respective reputations. Software does now exist to help automate these procedures (e.g. ScholarOne’s Reviewer Locator), but their role and usage and how these might affect who and how often reviewers invited remains largely unknown.

Editors wield supreme, executive power in the scholarly publishing decision-making process, rather than it being derived from a mandate from the masses. Because of this, scholarly publishing is inherently meritocratic (ideologically and perhaps in practice), rather than being democratic. Despite this, how editors attained their positions is rarely known, as are the motivations behind why some editors might start their own journal, write their own editorials or solicit submissions from other researchers. This is further complicated when conflicts might arise between the commercial interests or influence of a publisher (e.g. selling journals) and editorial concepts around academic freedom and intellectual honesty and integrity. There are around 33,100 active scholarly peer-reviewed English-language journals, each with their own editorial and publishing standards [2], emphasising the potential scale of this problem.

Editorial decisions are largely subjective and based on individuals and their relative competencies and motivations; this includes, for example, how they see their journal fit within the present and future research and publishing landscape as well as the perceived impact a paper might have both on their journal and on the research field. These biases are extremely difficult to conceptualise and measure and almost certainly always lacking in impartiality. Such editorial biases also relate to issues of epistemic diversity within the editorial process itself, which can lead to knowledge homogenisation, a perpetuation of the ‘Matthew effect’ in scholarly research [52, 53] and inequities in the diffusion of scientific ideas [54]. These issues are further exacerbated by the fact that editors often fail to disclose their conflicts of interest, which can be viewed as compromising their objectivity [55, 56], and the extent to which editors treat their reports seriously, as well as any dialogue between them and reviewers and authors [57]. For example, how an editor might decide to signal to authors which reviewer comments are more important to address and which can be overlooked and consequently, how authors might then deal with these. Just like questionable research practices or misconduct such as fraud, often these factors will remain invisible to peer review and the research community [58].

Journals and publishers can assist with these issues in a number of ways. For example, simply providing the name of the handling editor and any other editorial staff involved in a manuscript, including any other professional roles they have, any previous interactions they might have had with both reviewers and authors and the depth of evaluation they applied to a manuscript. However, such information could inadvertently lead to superficial judgements of research based more on the status of editors. Journals can also share data on their peer review workflows, including referee recommendations where possible [59]. The relationship of such recommendations to editorial decisions has currently only been performed at a relatively small scale for single journals [60, 61] and requires further investigation [62]. Disclosure of this information would provide not only great insight into editorial decisions and their legitimacy, but also be useful in improving review and editorial management systems, including based around training and support [6]. This could also be used to help to clarify what the conditions required in order to meet the quality criteria at different journals are, as well as whether authors are made fully aware of review reports and how these intersect with those criteria.

### Role of reviewers in peer review

It is known that, to various degrees, factors, such as author nationality, prestige of institutional affiliation, reviewer and nationality, gender, research discipline, confirmation bias and publication bias, all affect reviewer impartiality in various ways [63], with potential negative downstream consequences on the composition of the scholarly record, as well as for the authors themselves. However, this understanding of peer review bias is typically based on, and therefore limited to, available (i.e. published) data—usually at a small, journal-based scale—and not fully understood at a systems-level [37, 64]. These biases can range from subtle differences to factors that majorly influence the partiality of individuals, each one being a shortcut to decision-making that potentially compromises our ability to think rationally. Additional personal factors, such as life experiences, thinking style, workload pressures, psychography, emotional state, cognitive capacity, can all potentially influence reviewers, and almost certainly do. Furthermore, there remain a number of different additional complex and hidden social dimensions of bias that can potentially impact review integrity. For example, relationships (professional or otherwise) between authors and reviewers remain largely unknown—whether or not they are rivals or competitors, colleagues, collaborators or even friends/partners, each of which can introduce bias in a different way into peer review [9, 65, 66]. Finally, the relationship between journal policies relating to these factors and the practical application of those policies, and the consequences of such, still remains poorly understood.

The potential range of biases calls into question of what defines a ‘peer’ and our understanding of ‘expertise’. Expertise and the status of a peer are both incredibly multi-dimensional concepts, varying across research disciplines, communities, demographics, career stage, research history and through time. Yet the factors that prescribe both concepts remain often highly concealed, and both can ultimately affect reviewer and editorial decisions, for example, how reviewers might select which elements of an article to be more critical of, and subjective notions of, ‘quality’ or relevance. It is unclear whether or not reviewers ‘get better’ through time and experience, and whether the ‘quality’ of their reviewing varies depending on the type of journal they are reviewing for, or even form of research (e.g. empirical versus theoretical).

Often, there is a lack of distinction between the referee as a judge, juror and independent assessor. This raises a number of pertinent questions about the role of reviewer recommendations, the function of which varies greatly between publishers, journals and disciplines [5]. These expectations for reviewers remain almost universally unknown. If access to the methods, software and data for replication is provided, it is often unclear if reviewers are requested or expected to perform these tests individually or if the editorial staff are to do so. The fact that the assessment of manuscripts requires a holistic view, which requires attention to a variety of factors, including stylistic aspects or findings novelties, makes the task and depth of reviewing extremely challenging. It is also exceptionally difficult or impossible to review data once they have been collected, and therefore there is an inherent element in trust that methods and protocols have been executed correctly and in good faith. Exceptions do exist, largely from the software community, with both the *Journal of Open Research Software* and *Journal of Open Source Software* clearly requiring code review as part of their processes. While there is also a general lack of rewards/incentives that could motivate reviewers to embark in rigorous testing or replications, some journals do now offer incentives such as credits or discounts for future publications for performing reviews. However, how widespread or attractive these are for researchers and the potential impact they might have remains poorly known. Editors and journals have strong incentives to increase their internal controls, which they often informally outsource this effort to often uninformed reviewers.

Only recently, in the field of biomedicine, has there been any research conducted into the role and competencies of editors and peer reviewers [6, 67, 68]. Here, reviewers were expected to perform an inconsistent variety of multiple tasks including providing recommendations, addressing ethical concerns, assessing the content of the manuscript and making general comments about submitted manuscripts. While some information can be gained by having journals share data on the peer review workflows and decisions made by editors and the respective recommendations from reviewers, this will only paint an incomplete picture about the functional role of reviewers and how this variation in the division of labour and responsibility influences ultimate decision-making processes. While this can be functional to sharing editorial risk in the decision-making [69], it often undermines responsibility with negative implications on the legitimacy of the decision as it is perceived by authors [56].

The only thing close to a system-wide standard, that we are aware of, in this regard is the ‘Ethical Guidelines for peer reviewers’ from the Committee on Publication Ethics (COPE). At present, we have almost no understanding of whether or not authors and reviewers obligingly comply with such policies, irrespective of whether they actually agree with them or not. For example, how many reviewers sign their reports even during a blinded process and what the potential consequences of this (e.g. on reviewer honesty and integrity) might be or even the extent to which such anonymity is compromised [70]. There is an obligation here for journals to provide absolute clarity regarding the roles and expectations of reviewers and how their reviews will be used and to provide data on policy compliance through time.

One of the most critical ongoing debates in ‘open peer review’ regards whether or not blinding should be preferred as it offers justifiable protection, compared to the times when blinding encourages irresponsible behaviour during peer review [63, 70, 71]. For example, it is commonly cited that revealing reviewer identities could be detrimental or off-putting to early career researchers or other higher risk or under-represented communities within research due to offending senior researchers and suffering reprisals. Such reprisals could be either public or more subtle (e.g. future rejection of grant proposals or sabotage of collaborations). It has also recently been argued that a consequence of such blinding is concealing of the social structures that perpetuate such biases or inequities, rather than actually dealing with the root causes [72], and this reflects more of a problem with the ability for individuals within academia to abuse their status to the detriment of others [64]. However, the extent to which such fears are based on real and widespread events, or more conceptual or based on ‘anecdata’, remains largely unknown; a recent survey in psychology found that such fears are actually greatly exaggerated from reality [73], but such might not necessarily extrapolate to other research fields. Additionally, there is a long history of open identification at some publishers (e.g. PeerJ, BioMed Central) that could be leveraged to help assess the basis for these fears. There is also some evidence to suggest that blinding is often unsuccessful, for example in nursing journals [74]. Irrespective, any system moving towards open identities must remain mindful of these concerns and make sure such risks can be avoided. It remains to be seen whether even stricter rules and guidelines for manuscript handling, with ‘triple-blinded’ and automated systems can provide a better guard against both conscious and unconscious bias [75].

There are also critical elements of peer that can be exposed by providing transparency into the identity of reviewers [16, 76]. Presently available evidence on this remains often inconclusive, at the local scale, or often even in conflict as to what the optimal model for reducing or alleviating bias might be [43, 70, 77–81]. Simply exposing a name does not automatically mean that all identity-related biases are automatically eliminated; but it serves three major purposes:

- First, if reviewer identities are known in advance, we might typically *expect* them to be more critical and objective rather than subjective during the review process itself, as transparency in this case imposes at least partial accountability. With this, it can be examined as to whether this leads to higher quality reviews, lengthier reports, longer submission times, influence on reviewer recommendations and the impact this might have on research quality overall; factors that have been mostly overlooked in previous investigations of this topic. Journals can use these data to assess the potential impact these have on the cost and time management for peer review.
- Second, it means that some of the relationships and motivations of a reviewer can be inspected, as well as any other factors that might be influencing their decision (e.g. status, affiliation, gender). These can then be used to assess the uptake of and attitudes towards open identities, and whether there are systematic biases in the process towards certain demographics. More pragmatically for journals, these can then be compared to reviewer decline rates to streamline their invitation processes.
- Third, it means that if some sort of bias or misconduct does occur during the process, then it is easier to address if the identity of the reviewer is known, for example, by a third-party organisation such as COPE.

### Functionality and quality of peer review

Peer review is now almost ubiquitous among scholarly journals and considered to be automatically required and an integrated part of the publication process, whether it is functionally necessary or not. There is a lack of consensus about what peer review is, what it is for and what differentiates a ‘good’ review from a ‘bad’ review, or how to even begin to define review ‘quality’ [82]. This sort of lack of clarity can lead to all sorts of confusion among discussions, policies and practices. Research ‘quality’ is something that inherently evolves through time; for example, the impact of a particular discovery might not be recognised until many years after its original publication. Furthermore, there is an important distinction between ‘value’ and ‘quality’ for peer review and research; the former is a more subjective trait and related to the perception of the usage of an output, and its perceived impact, whereas the latter is more about the process itself as an intrinsic mark of rigour, validation or certification [83].

There are all sorts of reasons why this lack of clarity has transpired, primarily owing to the closed nature of the process. One major part of this uncertainty pertains to the fact that, during the review process, we typically have no idea what changes were actually made between successive versions. Comparison between preprints shared on *arXiv* and *bioRxiv* and their final published versions, for example, has shown that overall peer review seems to contribute very few changes and that the quality of reporting is similar [69, 84]. Assessment of the actual ‘value add’ of peer review remains difficult at scale, despite version control systems being technologically easy to implement [23, 85, 86], for example at the *Journal of Open Source Software*.

This problem is ingrained in the inherently diverse nature of the scholarly research enterprise, and thus peer review quality can relate to a multitude of different factors, e.g. rigorous methodological interrogation, identification of statistical errors and flaws, speed or turn-around of review, or strengthening of argumentation style or narrative [87]. Such elements that might contribute towards quality are difficult to assess in any formative way due to the inherent secrecy. We are often unable to discern whether peer reviews are more about form or matter, whether they have scrutinised enough to detect errors, whether or not they have actually filtered out ‘bad’ or flawed research, whether the data, software and materials were appropriately inspected, or whether replication/reproducibility attempts were made. This problem is reflected by the discussion above regarding the expected roles of reviewers. If research reports were made openly accessible, they could be systematically inspected to see what peer review entailed at different levels, and provide empirical evidence for its function. This could then also be used to create standardised peer review ‘check-lists’ to help guide reviewers through the process. Research and development of tools for measuring the quality of peer review are only in their relative infancy [82], and even then focused mostly on disciplines such as biomedicine [88].

It is entirely possible that some publishers have already gathered, processed and analysed peer review data internally to measure and improve their own systems. This represents a potentially large file drawer problem, as such information is only of limited use if only used for private purposes, or only made public if it enhanced the image or prestige of their journals. There are a number of elements of the peer review process that empirical data could be gathered, at varying degrees of difficulty, to better understand its functionality, including:

- Duration of the length of different phases of the process (note that this is not equivalent to actual time spent) [89, 90]
- Number of referee reports per article
- Length of referee reports
- Number of rounds of peer review per article
- Whether code, data and materials were made available during the review process
- Whether any available code, data or materials were inspected/analysed during the process
- The proportion of reviewers who decline offers to review and if possible, why they do
- Relative acceptance rates following peer review
- Who decides whether identities should be made open (i.e. the journal, authors, reviewers and/or editors), and when these decisions are made in the process
- Who decides whether the reports should be made open, when these decisions are made during the process, and what should be included in them (e.g. editorial comments)
- Proportion of articles that get ‘desk rejections’ compared to rejection after peer review
- Ultimate fate of submitted manuscripts
- Whether the journal an article was ultimately published in was the journal to perform the review (important now with cascading review systems)
- Whether editors assign particular reviewers in order to generate a specific desired outcome

These represent just some of the potential data sources that could be used to provide evidence for the key question of what peer review actually does and compare these factors through time, across and between disciplines and systematically. For example, it would be interesting to look at how peer review varies at a number of levels:

- Between journals of different ‘prestige’
- Between journals and publishers from across different disciplines
- Whether any differences exist between learned society journals and those owned by commercial publishers
- Whether peer review varies geographically
- Whether there are some individuals or laboratories who perform to an exceptional standard during peer review
- How all of these factors might have evolved through time

### Peer review and reproducibility

There are two core elements to examine here. First, if peer review is taken to be a mark of research quality, this raises the question of whether or not peer review itself should be reproducible; an issue that remains controversial. There is little current concrete evidence that it is, and research into inter-reviewer reliability (just one aspect of reproducibility) shows variable results [58, 91]. Second, peer review is currently limited in being physically able to reproduce experiments made, despite this being a core tenet of scholarship. Thus, the default is often to trust that experiments were performed correctly, data were gathered and analysed appropriately, and the results are reflective of this. This issue is tied to the above discussions regarding the expectation of reviewers as well as the function of peer review. Indeed, it remains critically unknown whether specialised reviewers (e.g. in methods, statistics) are used and actually apply their skills during the review process to test the rigour of performed research. There is potential here for automated services to play a role in improving reproducibility, for example, in checking statistical analyses for accuracy. However, increasing adoption of automated services during peer review is likely to raise even more questions about the role and function of human reviewers.

This is perhaps one of the main reasons why fraudulent behaviour, or questionable research practices, still enter the scholarly record at high proportions, even though peer review occurs [15, 92]. The Peer Reviewers’ Openness Initiative was a bold step towards recognising this [69, 91], in terms of increasing the transparency and rigour of the review process. However, it has not been widely adopted as part of any standardised review process and remains relatively poorly known and implemented. This is deeply problematic, as it means that reproducibility is something often considered post hoc to the publication process, rather than a formal requirement for it and as something tested by the review process. This has a number of consequences such as the ongoing and widespread ‘reproducibility crises’ [32]. Much of this could probably have been avoided if researchers were more cautious in conducting research and interpreting results, if incentives were aligned more with performing high-quality research than publishing in ‘high impact journals’ [84, 93, 94] and if peer review was more effective at ensuring reproducibility.

### Social and epistemic impacts of peer review

In terms of the influence of peer review subsequent to the formalised process itself, the actual impact it has on scientific discourses remains virtually unknown. Peer review is a bi-directional process, and the authors, editors, and reviewers all stand to gain from it as a learning experience and for developing new ideas. Not only is such learning potential highly variable across disciplines, but also is an incredibly difficult aspect to empirically measure. Little attention has been paid to the relationship between peer review as a mark of quality assurance and other post-publication forms of research evaluation. Recent research has documented the extent to which evaluation is based on criteria such as the journal impact factor [93], something which is decoupled from peer review. Indeed, the relationship between pre-publication evaluation and post-publication assessment has received virtually no attention, as far as we are aware, at either the individual, journal, publisher, discipline, institute or national levels. It is entirely possible that if we gained a deeper empirical understanding of peer review as a primary form of research evaluation, it could help to reduce the burden and impact of secondary systems for career advancement.

One potential solution to this has been an increasing push to publish review reports. However, similar to open identification, such a process creates a number of potential issues and further questions. For example, does knowledge that review reports will be publicised deter reviewers from accepting requests for review? And does this knowledge change the behaviour of reviewers and the tone and quality of their reports? This issue could go both ways. Some researchers, under the knowledge that their reports will be published, will strive to make it as critical, constructive, and detailed as possible; irrespective of whether or not their names are associated with it. Others, however, might feel that this can appear too combative and thus be more lenient with their reviews. Therefore, there are outstanding questions on how opening reports up can affect the quality, substance, length and submission time of review reports, as well as any associated costs. Such is further confounded by the fact that the record of public review reports will be inherently skewed based on the articles that are ultimately published and may exclude reviews for articles which remain rejected or ultimately unpublished.

Regarding many of the social issues we have described, care needs to be taken to distinguish between which biases/traits are intrinsic to peer review itself and which are passively entrained within peer review due to larger socio-cultural factors within research. For example, if a research community is locally centralised and homogeneous, this will be reflected in lower epistemic diversity during peer review; whereas the opposite may be true for more heterogeneous and decentralised research communities. It is imperative to understand not only the diversity of opinions that are being excluded in peer review, but also the consequences of epistemic exclusion. The totality of bias in human-driven peer review can likely never be fully eradicated, and it is unlikely that we will ever witness the implementation of a purely objective process. However, by assessing and contextualising them in as much depth as possible, we can at least acknowledge and understand the influences these have, and begin to systematically mitigate any potentially deleterious effects that such biases might have on peer review.

Furthermore, there is relatively little understanding of the impact of peer review on innovation. It has been previously claimed that peer review, as it is often employed, leads to conservatism through suppression of innovation or greater acknowledgement of limitations [45, 95], as well as ideological bias, but it is difficult to gauge the reality of this. If peer review leads to epistemic homogeneity due to its conservatism, this can have negative consequences on the replicability of research findings [96]. As such, it remains virtually unknown what the dynamic trade-off is between innovation and quality control; the former of which relies on creativity and originality, while the latter relies on consensus, accuracy and precision. Where is the magic point between rapid dissemination and slow and critical assessment? At some point along this spectrum, does peer review become redundant or functionally obsolete in its present forms? Available evidence shows that often peer review tends to fail to recognise even Nobel-quality research, often rejecting it outright and thus resisting the process of scientific discovery [97, 98]. Providing insight into these questions is critical, as it impacts our understanding of the whole ideology of peer review in advancing scholarship, as well as its ability to detect or assign value to ‘impactful’ research. This is complicated further by the fact that peer review is often seen as a solution to generate trust in results and used as a method to distribute academic capital and standing among different research communities [4, 99], while we remain with a very limited understanding of whether it has achieved its objectives as a filtering method [83]. Irrespective of what the process entailed at an article level, peer review still assigns an imprimatur, via ‘stamp of approval’ or endorsement over which knowledge enters the scholarly record and can thus be built upon.

### Beyond traditional peer review

As well as all of the above, which are more based around obtaining information from ‘traditional’ journal-coupled editorial peer review processes, there are now also a number of novel services that allow for different forms of peer review. Often these are platforms that tend to decouple peer review from journals in one way or another, making it more participatory or offering ‘post-publication’ either over preprints or final published versions of record [23, 85]. Previous research has shown that on some open commenting systems, user engagement tends to be relatively low for research articles [89, 100]. Thus, there is the existential question of how to overcome low levels of uptake for open participation (either on preprints or final-version articles). It seems that a critical element here is whether an open participatory process requires editorial control, if elements of it can be automated and to what extent ‘quality control’ over referee selection impacts the process, for example, does it make conflicts of interest more difficult to detect. There is no doubt that editors will continue to play a prominent role here in terms of arbitration, quality control, and encouraging engagement while fostering a community environment [76]. However, whether this can be done successfully within an open participatory framework remains to be seen; either with or without journals. One potentially disruptive element here is that of micro-publications, in which engagement is potentially less time consuming and this participation can be streamlined and a simpler task, thus potentially increasing reviewer uptake. However, this assumption relies on editors maintaining a similar role to their traditional function, and one remaining question is what impact would removing editorial mediation have on open participation.

Several innovative systems for interactive peer review have emerged in the last decades. These include the Copernicus system of journals, EMBO, eLife, and the Frontiers series. Here, peer review remains largely an editorially controlled process, but the process between reviewers and authors is treated more as a digital discussion, until some sort of consensus is usually reached to help guide an editorial decision. At present, it remains largely unknown whether this process is superior to the traditional organised unilateral series of exchanges, in the context of whether this process leads to a generally higher review quality or more frequent error detection. Logistically, it remains largely unknown whether this leads to a faster and more efficient review process overall, with potential consequences on the overall cost of managing and conducting peer review.

The principal reason why the World Wide Web was created and now exists was for the sharing of research results and articles prior to peer review (i.e. preprints), and either in parallel to or circumnavigating the slower and more costly journal-coupled review and communication processes [90, 101, 102]. However, this does not mean that preprints are the solution to all issues around peer review and scholarly publishing, especially as they are still regarded in different ways by different communities; something that undoubtedly requires further study [99]. With the recent explosion of preprints in the Life Sciences [103], a number of different services have emerged that ‘overlay’ peer review in one form or another on top of the developing preprint infrastructure [104], for example, biOverlay in the Life Sciences. However, the general uptake of such services appears to be fairly low [105]; most recently, this led to Academic Karma, a leading platform in this area, to shut-down (April 2019). In February 2018, the Prelights service was launched to help highlight biological preprints, and Peer Community In represents a service for reviewing and recommending preprints, both independent from journals. PREreview is another recently launched service that facilitates the collaborative review of preprints [106] The impact and potential sustainability of these innovative ‘deconstruction’ services, among others, is presently completely unknown. The fate of articles that pass through such a process also remains obscured; do they end up being published in journals too, or do authors feel that the review and communication process is sufficient to deem this unnecessary.

As well as services offering commenting functions on top of preprints, a number also exist for commenting on top of final, published versions of peer-reviewed articles. This includes services such as ScienceOpen and PubPub, as well as those that mimic the Stack Overflow style of commenting, including PhysicsOverflow, an open platform for real-time discussions between the physics community combined with an open peer review system, and MathOverflow, with both often considered to be akin to an ‘arXiv-2.0’. A system that sits in both this category and that of open pre-review manuscripts is that developed by F1000. This growing service is backed by big players including the Gates Foundation and Wellcome Trust [107]. Here, it works virtually the same as a traditional journal, except that submitted articles are published online and the subject to continuous, successive and versioned rounds of editorially managed open peer review. These services are all designed with the implication that review and publication should be more of a continuous process, rather than the quasi-final and discretised versions of manuscripts that are typically published today. There remains a large gap in our understanding of the motivations for people to engage, or not, with such platforms, as well as whether or not they lead to changes in the quality of peer review.

### Researcher attitudes towards [open] peer review

Within all of the ongoing innovations around peer review, shockingly little rigorous research has been conducted on researcher attitudes towards these changes. A recent survey (*n* = 3,062) provided a basis for understanding researcher perceptions towards changes around open peer review (OPR) [22]. Many of these problems must be framed against how researchers also view traditional forms of peer review, as well as against concurrent developments around preprints in different fields. With OPR now moving more into the mainstream in a highly variable manner, there remain a number of outstanding issues that require further investigation:

- Are the findings of levels of experience with and attitudes towards OPR reported in the survey results above consistent across studies?
- Which specific OPR systems (run via journals or third-party services) do users (within differing disciplines) most prefer?
- What measures might further incentivise uptake of OPR?
- How fixed are attitudes to the various facets of OPR and how might they be changed?
- How might shifting attitudes towards OPR impact willingness to engage with the process?
- What are attitudes to OPR for research outputs other than journal articles (e.g. data, software, conference submissions, project proposals, etc.)?
- How have attitudes changed over time? As OPR gains familiarity amongst researchers and is further adopted in scholarly publishing, do attitudes towards specific elements like open identities change? In what ways?
- To what extent are attitudes and practices regarding OPR consistent? What factors influence any discrepancies?
- Is an openly participatory process more attractive to reviewers, and is it more effective than traditional peer review? And if so, how many participants does it take to be as or more effective?
- Does openness change the demographic participation in peer review, for authors, editors, and reviewers?

## Discussion

This review of the limits to our understanding of peer review aimed to make clear that there are still dangerously large gaps in our knowledge of this essential component of scholarly communication. In Table 1, we presented a tabulated roadmap summarising peer review topics that should be researched (Table 1).

**Table 1:** Proposal for the future roadmap into peer review research

Topic	Topics recommended to be researched	Difficulty	Priority
Role of editors in peer review	Justifications for editorial decisions	Medium	High
	Factors that affect editorial quality, impartiality and their impact	Medium	High
	How editors select reviewers	Medium	Medium
	Impact of reviewer selection on relationships with editors and authors	Hard	Medium
	Editorial competencies and motivations for decisions	Medium	High
	Impact of decisions on epistemic diversity	Hard	High
	Editorial conflicts of interest and relationships with other parties	Easy	High
	Extent of editorial misconduct	Hard	High
	Influence of reviewer recommendations on editorial decisions	Medium	Medium
	Impact of editors’ careers on their scientific career	Medium	Low
Role of reviewers in peer review	Factors that affect reviewer impartiality and their impact	Medium	High
	Reviewer conflicts of interest and relationships with other parties	Easy	Medium
	Reviewer competencies and motivations	Easy	High
	Factors that affect inter-reviewer reliability	Medium	Medium
	Extent of peer review misconduct	Medium	High
	Expectations for reviewers	Easy	High
	Impact of incentives for reviewers	Medium	Low
	Conformation of reviewers to journal policies	Medium	Low
	Extent to which anonymity is compromised	Hard	Medium
	How do notions of expertise affect reviewer behaviour	Hard	Medium
	Impact of reviewing on scientific careers of reviewers	Medium	Low
Role of authors in peer review	Impact of author recommendations on reviews and reviewers	Medium	Medium
Functionality and quality of peer review	What peer review actually is and does	Medium	High
	How does peer review impact scientific discourse	Hard	High
	Relationship between peer review and journal quality	Medium	Medium
	Are there cases where peer review is redundant	Medium	Medium
	Reproducibility of peer review	Hard	High
	The development and impact of peer review standards	Medium	High
Social and epistemic impacts of peer review	Homogeneity and centralisation of reviewer pools	Medium	High
	Epistemic diversity of peer review	Hard	High
	Impact of peer review on innovation or conservatism	Hard	High
	Peer review as a vehicle for disseminating prestige	Hard	High
Type of peer review	Factors influencing the choice of peer review type	Medium	High
	Influence of peer review type on quality of review and potential misconduct	Medium	High
	Do micro-publications impact reviewer engagement	Medium	Low
	Is interactive peer review more effective	Medium	Medium
	How have/will preprints impact peer review	Medium	Medium
	Are overlay journals/services more effective	Medium	Medium
	Which OPR services do researchers prefer	Easy	Medium
	What measures can incentivise OPR	Medium	Medium
	Researcher attitudes towards OPR	Easy	High
	Researcher attitudes towards OPR for non-traditional outputs	Easy	Medium
	The impact of OPR on participant diversity	Medium	High
	The impact of blinding on biases and review quality	Medium	High
	Impact of open review reports	Hard	High
	Impact of review type on careers of reviewers	Medium	Medium

The difficulty levels primarily refer to the relative ease of obtaining empirical data for study, should such data even exist. The priority levels relate to their perceived impact on the future of peer review. Both are subjective estimates of the authors

Based on this roadmap, we see several high-priority ways in which to make immediate progress.

1. Peer Review Studies must be established as a discrete multi-disciplinary and global research field, combining elements of social sciences, history, philosophy, scientometrics, network analysis, psychology, library studies, and journalology.
2. To reach a global consensus on, and define a minimum standard for, what constitutes peer review; possibly as a ‘boundary object’ in order to accommodate field-specific variations [108]
3. To conduct a full systematic review of our entire global knowledge pool of peer review, so that the gaps in our knowledge can be more rigorously identified and quantitatively demarcated

These three key items should be then used as the basis for the systematisation of new research programmes, revealed by our analysis, combining new collaborations between researchers and publishers. In order to support this, it will require more funding, both from research funding bodies and publishers, both of whom need to recognise their respective duties in the stewardship and optimising the quality of published research. For this, a joint infrastructure for data sharing is clearly required as a foundation, based around a minimal set of criteria for standards and reporting. The ultimate focus of this research field should be fixed around the efficacy and value added by peer review in all its different forms. Designing a core outcome set would help to optimise and streamline the process to make it more efficient and effective for all relevant stakeholders.

All of the research items in this roadmap can be explored in a variety of ways, and at a number of different levels, for example, across journals, publishers, disciplines, through time and across different demographics. Views on the relevant importance of these issues may vary; however, in our opinion, based on the weights we would assign to their relative difficulty to address and level of importance, it would make sense to focus particularly on the following issues:

- Declaration of editorial conflicts of interests and documenting any editorial misconduct
- Expectations for, and motivations and competencies of, reviewer
- Researcher attitudes towards the various elements of [open] peer review

Taking a broad view, it is pertinent to tie our roadmap into wider questions surrounding reform in higher education and research, including ongoing changes in research funding and assessment. At present, peer review is systematically under-valued where most of it takes place—at academic institutions. Peer review needs to be taken seriously as an activity by hiring, review, promotion and tenure committees, with careful consideration given to any potential competitive power dynamics, particularly against earlier-career researchers or other higher risk demographics. Having it more valued at this level provides a strong incentive to learn how to do peer review correctly, while appreciating the deep complexities and diversity that surrounds the process. This includes establishing baseline knowledge and skills to form core competencies for those that are engaged in the review process so that they can fulfil their duties more appropriately [6]. This opening of the ‘black box of peer review’ will be critical for the future of an optimised peer review system, and avoiding any malpractice in the process.

There are several related elements to this discussion that we also elected not to discuss in order to maintain focus here. One of these is the issue of cost. Scholarly publishers often cite that one of the most critical things that they do is manage the peer review process—which is almost invariably performed as a voluntary service by researchers. Some estimates of the human time and cost do exist, with an estimate in 2008 putting the value of voluntary peer review services provided at around £1.9 billion per year [109] and that around 15 million hours are wasted through redundancy in the reject-resubmit cycle each year [110]. Together, these show that there is clear potential for improved efficiency in many aspects of peer review and which requires further investigation. Further information into the total financial burden of peer review might enable a cost-benefit analysis which could benefit all current stakeholders engaged in the future of peer review. Such could measure the relative benefits of quality control via peer review with the time and cost associated, as well as the impact of it preventing certain forms of knowledge entering scholarly discourses, and how this reflects epistemic diversity throughout the wider research enterprise.

## Conclusions

This article addressed unknowns within our current understanding of journal-coupled peer review. This represents a critical overview that is distinct from previous work, which has largely focused on what we can say based on the limited available evidence. Peer review is a diverse and versatile process, and it is entirely possible that we have missed a number of important elements. We also recognise that there are simply unknown unknowns (i.e. things we do not know that we do not know). Furthermore, the fact that peer review is not a mechanism isolated from the context but an essential part of a complex, evolving ecological system, which involves different entities interacting in the domain of scholarly research and communication, makes this challenge even more difficult. As such, there is scope for extending what we have done to other forms of peer review, including for grants and clinical trials [111, 112].

We hope here to have presented researchers with both a call to action and a roadmap for future research to progress their own research agendas as well as our communal knowledge of peer review by shining some light into the peer review box. Our effort was aimed to stimulate a more rational, less ideological approach and create the conditions for developing collaborative attitudes between all stakeholders involved in the scholarly communication system [76, 113]. In order to support this, we believe that we critically need a sustained and strategic programme of research dedicated to the study of peer review. This requires direct funding from both publishers and research funding bodies, and the creation of a shared, open data infrastructure [114]. Such could coalesce around, for example, the International Peer Review Congress [115].

This will help to ensure that state-of-the-art research employs similar vocabulary and standards to enable comparability between results within a cohesive and strategic framework. Substantial steps forward in this regard have recently been made by Allen et al. [40]. Such progress can also help us to understand which problems or deficiencies are specific to peer review itself, and so can be at least in principle improved through incremental or radical reforms, and which problems are nested within, or symptomatic of, a wider organisational or institutional context, and so requiring other initiatives to address (e.g. academic hypercompetition and incentive systems).

Our final wish is that all actors within the scholarly communication ecosystem remain cognizant of the limitations of peer review, where we have evidence and where we do not, and use this to make improvements and innovations in peer review based upon a solid and rigorous scientific foundation. Without such a strategic focus on understanding peer review, in a serious and co-ordinated manner, scholarly legitimacy might decline in the future, and the authoritative status of scientific research in society might be at risk.

## Data Availability

NA.
